# Applications, challenges and future directions of artificial intelligence in cardio‐oncology

**DOI:** 10.1111/eci.14370

**Published:** 2025-04-07

**Authors:** Francesco Ravera, Nicolò Gilardi, Alberto Ballestrero, Gabriele Zoppoli

**Affiliations:** ^1^ Department of Internal Medicine and Medical Specialties University of Genoa Genoa Italy; ^2^ IRCCS Ospedale Policlinico San Martino Genoa Italy

**Keywords:** artificial intelligence, cardio‐oncology, deep learning, electrocardiogram, imaging, machine learning

## Abstract

**Background:**

The management of cardiotoxicity related to cancer therapies has emerged as a significant clinical challenge, prompting the rapid growth of cardio‐oncology. As cancer treatments become more complex, there is an increasing need to enhance diagnostic and therapeutic strategies for managing their cardiovascular side effects.

**Objective:**

This review investigates the potential of artificial intelligence (AI) to revolutionize cardio‐oncology by integrating diverse data sources to address the challenges of cardiotoxicity management.

**Methods:**

We explore applications of AI in cardio‐oncology, focusing on its ability to leverage multiple data sources, including electronic health records, electrocardiograms, imaging modalities, wearable sensors, and circulating serum biomarkers.

**Results:**

AI has demonstrated significant potential in improving risk stratification and longitudinal monitoring of cardiotoxicity. By optimizing the use of electrocardiograms, non‐invasive imaging, and circulating biomarkers, AI facilitates earlier detection, better prediction of outcomes, and more personalized therapeutic interventions. These advancements are poised to enhance patient outcomes and streamline clinical decision‐making.

**Conclusions:**

AI represents a transformative opportunity in cardio‐oncology by advancing diagnostic and therapeutic capabilities. However, successful implementation requires addressing practical challenges such as data integration, model interpretability, and clinician training. Continued collaboration between clinicians and AI developers will be essential to fully integrate AI into routine clinical workflows.

## Graphical abstract

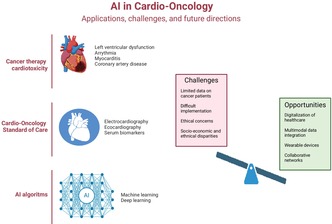

## 1 INTRODUCTION

In the last two decades, clinical oncology has experienced revolutionary advancements in the therapeutic management of patients living with cancer,^1^, ^2^, ^3^ leading to significant improvements in their outcomes. The development of novel drugs, often with very specific indications approved by regulatory agencies, has significantly increased the complexity of cancer care, with an unprecedented landscape of therapy‐related side effects and an increased need for a comprehensive management of cancer patients.^4^, ^5^

In this context, the relevance of cardio‐oncology has exponentially grown. Indeed, while the cardiotoxic effects of anthracyclines have been known for decades, the approval of agents targeting the human epidermal growth factor receptor 2 (HER2) and inhibitors of the vascular endothelial growth factor (VEGF) have significantly contributed to the development of cardio‐oncology as an independent medical discipline, incorporating skills and methodologies from clinical oncology, haematology, cardiology and internal medicine.^6^, ^7^ Overall, the spectrum of cardiovascular toxicities related to cancer treatment is highly heterogeneous, spanning from valvular toxicities to electrophysiologic, pericardial or thrombotic complications, all potentially leading to acute or chronic heart failure (HF).^6^, ^7^ More recently, the expansion of targeted therapies and the advent of immunotherapy have further increased the complexity of cardiac therapy‐related toxicities, with a wide range of novel side effects such as immune‐related myocarditis.^8^

The development and implementation of comprehensive guidelines by the European Society of Cardiology (ESC) represent a significant milestone in this field,^9^ further acknowledging the centrality of a comprehensive approach to cancer patients. Despite the relevance and the undoubted benefit resulting from the development of such guidelines, current approaches to the prevention and management of cardiotoxicity in patients with cancer are burdened by several limitations and unmet needs. While the intention of the ESC guidelines is to promote early detection of cardiotoxicity allowing prompt interventions, their recommendations for routine screening, even in low‐risk patients, have raised concerns about the potential for unnecessary and/or unfeasible testing and the associated burden for national health systems, with particular regard to the extensive echocardiogram‐based screening and monitoring programs suggested by the Society.^10^ Furthermore, current strategies used to stratify and treat cancer‐related cardiovascular toxicities, while based on established guidelines, may not be fully optimized for the diverse spectrum of anticancer treatments and the associated side effects.^9^

**FIGURE 1:**
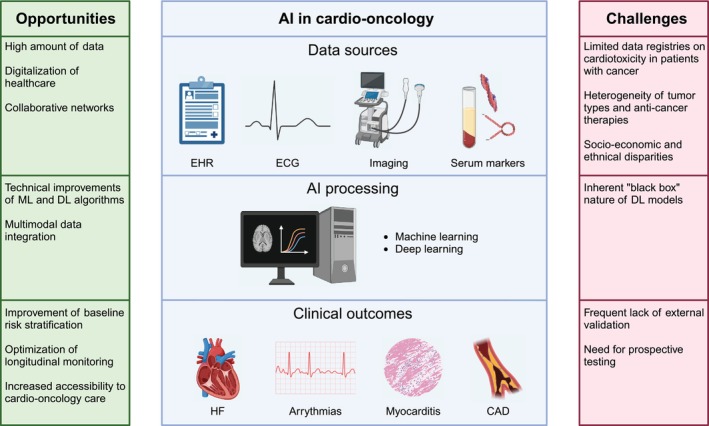
Overview of the opportunities and challenges of AI implementation in cardio‐oncology. CAD, coronary artery disease; DL, deep learning; EHR, electronic health records; HF, heart failure; ML, machine learning.

Artificial intelligence (AI), with its unique capacity to analyse massive datasets and discern subtle, complex patterns often missed by traditional analytical methods, presents a powerful means to overcome these limitations.^11^ AI's ability to integrate and analyse multiple data sources, including electronic clinical data, imaging and wearable sensor data, offers unprecedented opportunities to improve the accuracy and efficiency of cardio‐oncology practices, refining existing risk stratification models, enhancing diagnostic capabilities, and informing the development of more effective and personalized treatment strategies. Numerous studies have indeed demonstrated AI's ability to significantly improve the diagnostic and predictive capabilities of electrocardiograms (ECGs), cardiovascular imaging and cardiac biomarkers in nononcologic populations.^12^ While the potential benefits of AI are readily apparent in the cardio‐oncology setting, research specifically evaluating AI's impact on cardiotoxicity management in cancer patients remains limited, hindering the generalizability of current findings.^13^, ^14^, ^15^ This review critically examines the current evidence of AI applications in cardio‐oncology, focusing on studies conducted on patients with solid or blood tumours, and highlighting the technology's potential to address existing challenges along with its limitations and caveats (Figure 1). In particular, we describe how AI can support clinicians by optimizing the use of tools currently available in the standard cardio‐oncology practice, from clinical data available in electronic health records (EHRs) to ECG, noninvasive imaging, and circulating serum biomarkers, with a further spotlight on digital health technologies.

## 2 GENERAL PRINCIPLES OF ARTIFICIAL INTELLIGENCE

Several works have extensively reviewed the principles and implications of AI in medicine.^11^ Essentially, the application of AI in the healthcare setting heavily relies on two different core architectures, namely machine learning (ML), where algorithms learn patterns from data without the need for explicit programming, and deep learning (DL), a subset of ML that uses artificial neural networks (ANNs) with multiple layers (hence ‘deep’) to extract complex features from data. The increased complexity of DL allows to handle high‐dimensional data and learn intricate patterns without human supervision, performing generally better than traditional ML, either under or without supervision.^16^ Derivations of DL include convolutional neural networks (CNNs), primarily used for image analysis^17^ and particularly useful in the cardiovascular setting, recurrent neural networks (RNNs), primarily used for sequential data analysis,^18^ Long Short‐Term Memory networks, a type of RNN particularly effective at handling long‐range dependencies in sequential data,^19^ and Generative Adversarial Networks, typically used for generating synthetic medical data to augment training datasets or create realistic simulations for medical training.^20^

The application of AI has been explored in several fields of healthcare, spanning from outcome prediction to medical imaging, diagnostics, treatment planning and personalization, drug discovery and development, robotic surgery and endoscopy.^11^, ^21^ In this context, Natural language processing represents a self‐standing niche and is increasingly used in the medical setting to analyse unstructured clinical text data, optimize clinical documentation and assist writing.^22^

Although each different discipline comes with specific requirements and characteristics, the development of clinically useful AI algorithms faces common challenges across the different settings. In particular, high‐quality, well‐annotated data is essential for training AI models, as they can inherit biases present in the input data, leading to inaccurate or unfair predictions.^23^ Indeed, verifying the robustness of AI models through rigorous external validation processes is a crucial step to attest their potential utility in clinical practice.^23^, ^24^ Moreover, the inherent ‘black box’ nature of many DL models hinders their interpretability.^25^ Finally, the use of AI in healthcare raises important regulatory and ethical considerations related to data privacy, patient safety and algorithmic accountability.^26^

## 3 AI AND ELECTRONIC HEALTH RECORDS IN CARDIO‐ONCOLOGY

The digitalization of healthcare systems has allowed for the generation and storage of massive amounts of data, creating unprecedented opportunities for transformative advancements in the medical setting. EHRs serve as a rich source of longitudinal patient data, encompassing a vast array of information, from demographics to clinical histories, laboratory results, imaging, medication records and even genomic data. This wealth of information, previously largely inaccessible for comprehensive analysis without time‐ and resource‐consuming manual review, provides a unique and powerful resource for training and validating AI algorithms designed to improve cardio‐oncology practices. Several studies have indeed explored the use of AI in the assembly of risk predictors of cardiotoxicity in patients with cancer. In a work by Hou et al.,^27^ a patient–patient network analysis methodology was developed to identify cancer patients at high risk of developing cancer treatment‐related cardiac dysfunction (CTRCD), including atrial fibrillation (AF), coronary artery disease (CAD), HF, myocardial infarction or stroke occurring after the start of cancer therapy. Leveraging a large, institutional EHR database comprising 4632 patients with solid or blood cancer, the authors constructed patient–patient networks based on cosine similarity scores calculated from a wide array of clinical variables, including demographics, laboratory results, echocardiogram data and cancer treatment details. Using a topology‐based K‐means clustering approach, four distinct patient subgroups with different cardiovascular risk profiles were identified. Notably, serum levels of NT‐proBNP and Troponin T emerged as significant predictors of mortality. In parallel, Zhou et al.^28^ developed several predictive models for CTRCD in a cohort of 4309 cancer patients treated with chemotherapy and/or radiotherapy. Chemotherapy regimens mostly comprised anthracyclines, cyclophosphamide and trastuzumab. The study integrated echocardiographic and laboratory variables to train and evaluate five different ML algorithms (k‐nearest neighbours, logistic regression, support vector machine, random forest (RF) and gradient boosting) across three feature sets (laboratory data only, echocardiographic data only and combined data). The models achieved high accuracy in predicting six cardiovascular outcomes, with the combined feature set generally outperforming individual ones. Notably, the logistic regression model achieved the highest accuracy across the majority of outcomes. More recently, in a study by Al‐Droubi et al.,^29^ ML algorithms were applied to develop predictive models for identifying cancer patients at high risk of developing CTRCD. Using a large dataset of de‐identified EHRs encompassing patients with breast cancer, kidney cancer, B‐cell lymphoma and those receiving immunotherapy, the authors trained and tested RF and ANN models. Both models demonstrated high accuracy in assessing CTRCD risk, with ANN outperforming RF in this setting.

Generally speaking, these case examples highlight how there is no ‘catch‐them‐all’ algorithm in mathematical learning, but rather, a family of methods helping clinicians to identify novel solutions to clinical problems.

## 4 AI AND ELECTROCARDIOGRAPHY IN CARDIO‐ONCOLOGY

ECG is a foundational tool in cardio‐oncology, from initial risk assessment to ongoing monitoring and long‐term surveillance, providing a readily accessible and cost‐effective method for assessing cardiac function and detecting abnormalities in patients undergoing cancer treatment. The integration of AI with ECG holds significant potential for recognizing subtle patterns and abnormalities in cardiac waveforms associated with subclinical pathological processes, ultimately increasing its diagnostic and predictive power. Numerous works, reviewed in,^30^, ^31^ have indeed demonstrated the effectiveness and potential clinical utility of AI applied to ECG images (AI‐ECG) in the assessment of a wide range of cardiac conditions. Several studies have focused on the application of AI‐ECG for the detection and/or prediction of structural heart diseases, with particular regard for left ventricular dysfunction (LVD), a common and serious side effect of widely used anticancer drugs such as anthracyclines.^6^ Notably, Attia et al. developed a DL algorithm based on CNN to diagnose asymptomatic LVD using ECG data from more than 40,000 patients.^32^ The algorithm achieved high accuracy in the testing cohort comprising 52,870 patients, maintaining a robust performance in two distinct validation cohorts.^33^, ^34^ Furthermore, the same research group demonstrated in a randomized clinical trial that the prospective application of the previously developed AI‐ECG algorithm allows for a significantly higher detection rate of LVD in subjects undergoing ECG testing in the setting of routinary primary care compared to the standard clinical workflow.^35^ Among patients with positive AI‐ECGs, those in the interventional arm received significantly more echocardiograms compared to those in the control arm, often resulting in the update of their medical treatment.

AI‐ECG has also shown promise in the early diagnosis and prediction of arrhythmias, with particular regard to AF and long QT syndrome (LQTS), relatively frequent side effects of several anticancer treatments such as inhibitors of CDK4/6 kinase or Bruton tyrosine kinase.^36^, ^37^, ^38^ Similarly to the approach used for the detection of asymptomatic LVD, Attia et al. developed an AI algorithm able to accurately detect subclinical AF in patients with normal sinus rhythm using ECG data collected from 180,922 patients,^39^ while Bos et al. report that AI‐ECG outperforms standard corrected QT interval in assessing LQTS even in patients with ECG‐concealed syndrome, providing accurate indications on their genotypic status.^40^

Other works showed promising results of different AI algorithms in the early detection of myocardial hypertrophy, ischemic disease, cardiomyopathy and pulmonary hypertension, reporting good results in terms of accuracy for the specific condition investigated.^41^, ^42^

Even though the vast majority of works regarding the use of AI‐ECG involves nononcologic patients, an increasing number of studies tailored on patients with solid or blood tumours has been designed over the last years (Table 1). In one of the first investigations focused on the assessment of cardiotoxicity in patients with cancer, Güntürkün et al. developed an AI‐ECG model predictive of LVD in a large population of adult survivors of childhood cancer exposed to radiation and/or anthracycline‐based therapy, reporting significantly higher accuracy of the AI‐ECG model compared to standard clinical predictors of cardiomyopathy.^43^ Jacobs et al. applied a CNN‐based model to detect LVD upon ECG features in patients with breast cancer receiving anthracyclines, reporting high accuracy even using different thresholds of LVEF for the definition of LVD.^44^ In parallel, Yagi et al. investigated the potential of AI‐ECG for predicting the risk of LVD in patients with either solid or blood cancer undergoing anthracycline‐based chemotherapy.^45^ In particular, by applying a transfer learning approach, the authors updated an AI model previously developed to predict asymptomatic LVD in the primary care setting^24^ using a training set of patients with matched ECG and echocardiogram performed before the start of anthracyclines chemotherapy. Despite the relatively low numerosity of the new training set (*N* = 317), the AI model effectively stratified LVD risk upon ECG features in a testing cohort comprising more than 1000 patients, independently of known risk factors for cardiovascular diseases as well as of tumour type, sex, baseline LVEF and initial anthracycline dose, significantly improving the accuracy of standard clinical predictors of cardiovascular disease. Finally, in a recent work by Oikonomou et al., the authors assessed the AI‐ECG performance in the evaluation of LVD in patients with breast cancer or non‐Hodgkin lymphoma treated with anthracyclines and/or trastuzumab.^46^ Similarly to Yagi et al.,^45^ the AI‐ECG model assessed on baseline ECG was able to stratify patients upon the risk of overall CTRCD and LVEF reduction, with patients having a positive baseline AI‐ECG screen bearing a 3.4‐fold and 4.9 higher risk of developing CTRCD and EF <50% compared to those with a negative screening, respectively. Interestingly, the authors reported that the longitudinal monitoring through AI‐ECG of sequential ECG performed *per* clinical practice showed dynamic changes in the AI‐ECG probability anticipating the occurrence of CTRCD, supporting a biologically meaningful association between ECG features captured by the AI model and the cardiac structural modifications occurring during cardiotoxic treatment. This was further supported by the significant association of AI‐ECG probability of LVD and the cardiac global longitudinal strain (GLS) assessed by standard echocardiography reported in the same work. Notably, this association was maintained even in patients with preserved LVEF.

**TABLE 1:** Articles investigating the implementation of AI to ECG in the cardio‐oncology setting.

Type of cancer	Type of anticancer treatment	AI model	Clinical purpose	Endpoints	Results	Sample size	Reference
Paediatric tumours	Anthracyclines radiation	Extreme Gradient Boosting	Prediction of cardiotoxicity	LVEF <50% and/or LVEF drop ≥10%	Sensitivity: 76% Specificity: 79% AUC: .87	1217	^43^
Breast cancer	Anthracyclines	CNN	Detection of cardiotoxicity	LVEF <50% LVEF <35%	AUC: .93 AUC: .94	703	^44^
Blood and solid tumours	Anthracyclines	CNN	Prediction of cardioxicity	LVEF <53% and/or LVEF drop >10%	HR: 2.66 for high AI risk patients	1011	^45^
Breast cancer Non‐Hodgkin lymphoma	Anthracyclines Trastuzumab	CNN	Prediction of cardioxicity	Cardiomyopathy and/or HF and/or LVEF <50% LVEF <50% LVEF <40%	HR: 3.55 for high AI risk patients HR: 4.88 for high AI risk patients HR: 13.52 for high AI risk patients	1550	^46^
Chronic lymphocytic leukaemia	Unspecified treatment Bruton tyrosine kinase inhibitors	CNN	Prediction of AF	Incident AF	HR: 3.9 for high AI risk patients HR: 2.8 for high AI risk patients	754 220 (110 with ECG within 1 year)	^47^

Abbreviations: AF, atrial fibrillation; AUC, area under the curve; CNN, convolutional neural networks; HR, hazard ratio; LVEF, left ventricular ejection fraction.

Concerning the prediction of arrhythmias in patients with cancer, Christopoulos et al.^47^ applied an AI‐ECG model previously developed in a noncancer population^39^ to predict the occurrence of AF in patients with a new diagnosis of chronic lymphocytic leukaemia, reporting a good risk stratification and providing complementary information with more standard clinical predictors of AF.

## 5 AI AND IMAGING IN CARDIO‐ONCOLOGY

Noninvasive imaging plays a pivotal role in the baseline risk assessment and longitudinal monitoring of patients at risk of cardiovascular events starting cardiotoxic chemotherapy. Transthoracic echocardiography (TTE) is the preferred initial imaging modality for assessing cardiac function and is recommended in all patients at high or very high cardiovascular risk before the start of chemotherapy and in all patients starting cardiotoxic treatments, such as anthracyclines or HER2‐targeting agents. Overall, TTE allows for a comprehensive evaluation of left and right ventricular function, chamber enlargement, ventricular hypertrophy, regional wall motion irregularities, diastolic function, valvular heart disease, pulmonary arterial pressure and pericardial disorders. Currently, the main criteria for defining CTRCD rely on the decrease of LVEF and/or relative alterations in GLS.^9^ Other imaging techniques, such as cardiac magnetic resonance (CMR) or nuclear imaging, are considered secondary modalities to be utilized when TTE yields poor image quality or if further characterization of the myocardium is required.^9^ Conversely, computed tomography (CT) scan is often performed in cancer patients as a part of their oncology care for screening, staging or radiotherapy planning and can be leveraged for the assessment of cardiovascular risk indicators such as coronary artery calcifications (CAC).^48^

To date, several works have explored the use of AI algorithms to refine the use of TTE in the cardiovascular setting, reporting good diagnostic accuracy and predictive potential. Zhou et al.^49^ used a ML approach, termed Shape Regression Machine (SRM),^50^ for segmenting the left ventricular endocardium in 2D B‐mode echocardiograms. SRM uses image‐based boosting ridge regression to model shape deformations efficiently, providing rapid and accurate detection critical for AI‐enhanced echocardiographic applications. In parallel, Sengur et al.^51^ investigated the application of support vector machine ensembles for diagnosing valvular heart disease through Doppler echocardiography. The study demonstrated that ensemble techniques like boosting and bagging significantly enhance diagnostic accuracy and reliability of echocardiography, supporting the role of AI algorithms in aiding clinical decision‐making and advancing precision in cardiology. Tabassian et al.^52^ developed a ML framework combining unsupervised statistical modelling (principal component analysis) and a supervised classifier (distance‐weighted k‐nearest neighbour) to analyse spatiotemporal patterns of left ventricular strain, strain rate and velocity during rest and exercise echocardiography. This approach significantly improved the identification of HF with preserved ejection fraction by detecting subtle abnormalities in myocardial deformation. In the context of cardio‐oncology (Table 2), Cheng et al. investigated the potential of AI applied to echocardiographic images to identify GLS features predictive of LVEF decline in a cohort of 248 breast cancer patients receiving doxorubicin chemotherapy.^53^ Applying ML algorithms to echocardiographic images collected at baseline and during the course of treatment, the authors were able to forecast LVEF decline. Notably, the correlation between initial AI‐refined GLS characteristics and subsequent LVEF declined over time, with stronger associations closer to treatment initiation and weaker correlations at 12 and 24 months. Mid‐septal and anteroseptal left ventricular segments, particularly in the circumferential and longitudinal dimensions, emerged as key predictive regions of cardiotoxicity.

**TABLE 2:** Articles investigating the implementation of AI to noninvasive imaging in the cardio‐oncology setting.

Type of cancer	Type of anticancer treatment	Imaging technique	AI model	Clinical purpose	Endpoints	Results	Sample size	Reference
Breast cancer	Anthracyclines	TTE	ML	Prediction of cardiotoxicity	Correlation between cardiac strain features and LVEF decline	R between cardiac strain features and LVEF decline: at baseline and 4 months = .50, at 12 months = .30, at 24 months = .24	248	^53^
NA	Anthracyclines	TTE	DL	Automated GLS assessment	Feasibility IRC	Feasibility: 98% IRC EchoGo—TomTec = .57 IRC EchoGo—QLAB = .71	52	^58^
Solid tumours	Anthracyclines HER2 targeting agents VEGF inhibitors RAF and MEK inhibitors Chest radiotherapy	TTE	CNN	LVEF assessment by oncology staff	Accuracy of LVEF assessment	Cardiologist = .94 Senior oncologist = .91 Junior oncologist = .92 Oncology nurse = .89	115	^60^
Subjects at risk for lung cancer	NA	CT scan	CNN	Prediction of cardiovascular mortality based on automated CAC score	Cardiovascular disease incidence Cardiovascular disease mortality	OR = 1.12 (for continuous values) OR = 1.05 (for continuous values)	12,332	^63^
Breast cancer	Radiotherapy	CT scan	CNN	Reliability of automated CAC and TAC assessment	Automated CAC assessment reliability Automated TAC assessment reliability	CAC reliability Netherlands = .85 TAC reliability Netherlands = .98 CAC reliability Singapore = .90 TAC reliability Singapore = .99	120 (Netherlands cohort) 120 (Singapore cohort)	^64^
Breast cancer	Radiotherapy	CT scan	CNN	Prediction of cardiovascular disease from automated CAC score	Incidence of fatal and nonfatal cardiovascular diseases	HR [CAC = 1–10] = 1.1 HR [CAC = 11–100] = 1.8 HR [CAC = 101–400] = 2.1 HR [CAC > 400] = 3.4	15,915	^65^
Subjects at risk for lung cancer	NA	CT scan	CNN	Detection of cardiovascular disease Prediction of cardiovascular mortality	Any cardiovascular abnormality reported in the CT screening exam or death for cardiovascular disease Death for cardiovascular disease	AUC = .871 AUC = .768	11,903	^66^

Abbreviations: AUC, area under the curve; CAC, coronary artery calcifications; CNN, convolutional neural networks; CT, computed tomography; DL, deep learning; GLS, global longitudinal strain; HR, hazard ratio; IRC, inter‐reader correlation; LVEF, left ventricular ejection fraction; ML, machine learning; OR, odds ratio; TAC, thoracic aorta calcifications; TTE, transthoracic echocardiography.

Another key aspect of AI application to echocardiography is the automatization of image acquisition processes, reducing the inter‐individual variability and expanding the accessibility to ultrasound records. Knackstedt et al.^54^ investigated the use of a fully automated software, termed AutoLV, which utilizes ML algorithms for the automated assessment of LVEF and GLS from 2D echocardiographic images, reporting high feasibility and a strong agreement with manual and visual assessments, while Cai et al.^55^ developed MMnet, a hybrid DL and ML model that automates the grading of diastolic function using echocardiographic parameters. The model evaluates key parameters, including mitral E and A wave velocities, septal and lateral e’ velocities, tricuspid regurgitation velocity, LVEF and left atrial end‐systolic volume, from 2D grey‐scale, pulse‐wave and tissue Doppler images. By integrating these features, the model achieves precise and efficient diastolic function grading, supporting the effectiveness of AI in enhancing echocardiographic diagnostics with high accuracy and clinical applicability. Zhang et al. developed and validated a fully automated pipeline for interpreting TTEs, leveraging DL and computer vision models.^56^ In particular, the authors aimed to automate key tasks in echocardiographic analysis, including view classification, chambers' image segmentation, quantification of cardiac structure and function, as well as disease detection, spanning from hypertrophic cardiomyopathy to cardiac amyloidosis and pulmonary arterial hypertension. Using CNNs trained on over 14,000 echocardiograms, the algorithm achieved high accuracy in identifying echocardiographic views and showed high consistency with manual measurements for parameters like left ventricular mass and LVEF. The study also included a sub‐analysis focused on 152 patients with HER2‐positive breast cancer who were receiving trastuzumab or pertuzumab, demonstrating the potential for tracking cardiotoxicity in cancer patients by accurately measuring GLS.^56^ Similarly, Ouyang et al. developed a DL algorithm, named EchoNet‐Dynamic, able to perform automated frame‐level segmentation of the left ventricle and employing spatiotemporal convolutions for LVEF prediction across multiple cardiac cycles. Trained on a dataset of 10,030 echocardiograms, EchoNet‐Dynamic demonstrated high segmentation accuracy (Dice coefficient .92) and a mean absolute error of 4.1% in LVEF estimation.^57^ Notably, Hanif et al. addressed the issues of inter‐reader and inter‐vendor variability in GLS assessment in the cardio‐oncology setting by evaluating TTE performed in patients treated with anthracycline‐based chemotherapy.^58^ In particular, an AI software named EchoGo Core was compared with conventional TTE softwares (TomTec and QLAB) in the assessment of GLS in standard 2‐ and 4‐chamber apical views, showing minimal bias and elevated feasibility, with a 98% success rate.

Overall, these results pave the way for increasing the accessibility to TTE, reducing the amount of expertise required for performing such exams. To this regard, Narang et al. showed that a DL‐integrated ultrasound scanner could guide novices in acquiring high diagnostic quality TTE,^59^ while Papadopoulou et al. investigated the use of AI‐enabled handheld ultrasound devices (HUDs) to assess LVEF in patients with cancer.^60^ The study evaluated 115 patients undergoing chemotherapy, comparing LVEF measurements by oncology staff not proficient in the use of echocardiography using AI‐assisted HUDs versus standard TTE performed by trained cardiologists. The AI algorithm demonstrated good diagnostic accuracy for detecting impaired LVEF, with sensitivity and specificity comparable to those of TTE proficient users, along with high inter‐observer reproducibility and test–retest reliability. These findings suggest that AI‐enabled HUDs could enhance point‐of‐care cardiac evaluations in oncology, facilitating early identification of cardiotoxicity and streamlining clinical workflows by enabling rapid assessment when proficient echocardiography users are not immediately available.

In the context of second‐level exams, several authors have applied AI algorithms to optimize the use of CMR, CT scan and nuclear imaging in cardiovascular care. Concerning CMR, ML and DL algorithms have been used to accelerate image acquisition, automate cardiac function assessment, characterize cardiac tissue features such as myocardial fibrosis or edema, and perform image denoising.^61^, ^62^ Similar results have been reported in the setting of nuclear imaging, indicating the potential of AI in improving the evaluation of myocardial perfusion, particularly relevant in the context of myocarditis caused by immune checkpoint inhibitors.^15^ Concerning CT scan, AI has been used to automatically assess CAC from CT images obtained *per* clinical practice in standard oncology care. Stemmer et al. and applied a ML approach to automatically assess CAC from CT scans collected from a cohort of more than 12,000 subjects with a history of heavy smoking at risk of lung cancer.^63^ Gernaat et al. investigated the use of a CNN algorithm for the automated assessment of CAC and thoracic aorta calcifications (TAC) in breast cancer patients who underwent CT scan for radiotherapy planning, reporting high reliability for the evaluation of both CAC and TAC.^64^ Similarly, Gal et al. used a DL algorithm for the automatic quantification of CAC from CT scans obtained from over 15,000 patients with breast cancer scheduled to receive radiotherapy, reporting a strong association between automatically calculated CAC score and cardiovascular risk.^65^ Notably, Chao et al. used low dose CT scan performed for lung cancer screening to identify patients with cardiovascular disease and to predict cardiovascular mortality, reporting areas under the curve of .87 and .77, respectively.^66^

## 6 AI AND CIRCULATING MARKERS OF CARDIOTOXICITY IN CARDIO‐ONCOLOGY

To date, there is limited evidence regarding the use of cardiac serum biomarkers in cancer patients starting antineoplastic treatment. The assessment of cardiac troponins and natriuretic peptides is currently recommended for baseline risk stratification, in the case such markers are used throughout patients' follow‐up for their longitudinal monitoring.^9^ However, the low number of studies investigating their predictive value in the context of CTRCD limits their relevance in this setting.^9^ Consistently with these knowledge gaps, no study has investigated the implementation of AI specifically addressed to optimize the assessment of cardiac enzymes in the cardio‐oncology setting so far. However, several works have explored the use of AI in conjunction with cardiac troponins or natriuretic peptides in the context of CAD^67^ and HF,^68^ respectively, with strong indications of the value of AI in aiding clinical decision‐making. Indeed, three distinct ML algorithms aimed at the detection of non‐ST elevated myocardial infarction have been developed and validated in large nononcologic patient cohorts, demonstrating significantly higher accuracy compared to standard clinical pathways used for the stratification of patients presenting at the emergency room with suspect of acute coronary syndrome (ACS).^69^, ^70^, ^71^ At the same time, using a ML algorithm combining NT‐proBNP concentration and clinical variables associated with acute HF, Lee et al. developed and validated a tool that significantly outperforms NT‐proBNP measurement alone, even within different patients' subgroups.^72^ These results are particularly relevant and can have a significant impact on cardio‐oncology, especially considering the parallel technological advancements concerning the use of wearable devices. Notably, Sengupta et al. assessed the feasibility of a wrist‐wearable transdermal sensor for the monitoring of patients hospitalized with ACS.^73^ In particular, the authors trained a DL model to identify elevated high‐sensitivity cardiac troponin‐I in patients hospitalized with ACS, externally validating the algorithm with standard laboratory assessment of cardiac troponin, echocardiography and angiography. While this is a preliminary feasibility study conducted in a small cohort of hospitalized patients, these results pave the way for the implementation of remote monitoring strategies for the intensive follow‐up of patients with high cardiovascular risk. Moreover, such technological implementations may be particularly useful for conditions where cardiac troponins are capital diagnostic criteria, such as immune‐related myocarditis.^9^

## 7 AI AND DIGITAL HEALTH IN CARDIO‐ONCOLOGY

Digital health technologies offer considerable potential to transform healthcare improving access to treatment, enhancing patient monitoring and facilitating communication between healthcare providers and patients. Telemedicine, wearable sensors and online resources can expand access to specialized cardio‐oncology services, particularly for patients in underserved areas. Keppel et al. have reviewed the potential of telehealth and AI to address healthcare disparities in cardio‐oncology, particularly concerning access to care for patients in rural communities.^74^ To this regard, AI‐enhanced chatbots can facilitate the collection of patient‐reported outcomes, such as symptom severity and medication adherence,^75^ while an improved access to health information through online resources, digital platforms and mobile applications can reinforce patients' education and adherence to treatment and follow‐up.^76^ Moreover, wearable sensors, such as smartwatches and fitness trackers, offer continuous streams of physiological data, including heart rate, activity levels, sleep patterns, ECG waveforms, and, potentially, even information regarding circulating markers of disease.^73^, ^77^ The application of AI algorithms to this continuous stream of multimodal data can be used to create sophisticated monitoring systems that generate timely alerts to patients and healthcare providers regarding potential complications, allowing for prompt interventions and improved management of cardiotoxicity.

## 8 CURRENT CHALLENGES AND FUTURE DIRECTIONS

The integration of AI into cardio‐oncology holds significant promise for enhancing patient care, but the path forward is complex and requires sustained effort. Currently, the body of research specifically involving cancer patients is limited, revealing a substantial gap in evidence that needs to be addressed.^78^, ^79^ Indeed, while there is an abundance of datasets focused on either cancer or cardiovascular conditions independently, fewer combine these domains in a way that captures the complexity and interplay of these coexisting conditions. This lack of integrated data hinders the development and training of AI models capable of addressing the unique challenges faced by cardio‐oncology patients. As a result, most AI algorithms rely on noncomprehensive datasets, which may fail to represent the full spectrum of clinical scenarios in this specialized field, ultimately limiting the generalizability and reliability of these technologies in real‐world applications. Moreover, studies carried out in oncological settings lack external validation and prospective testing, which are essential for establishing the robustness and clinical utility of AI algorithms in this specialized context. Ethical concerns around algorithmic bias must be addressed as well. If training datasets are not representative of diverse populations, AI models risk propagating existing healthcare disparities.^80^, ^81^ To overcome these challenges, the formation of large collaborative networks, including clinicians, technologists and policymakers, and the initiation of new, well‐designed studies are crucial steps forward.^78^, ^79^, ^82^ Another critical aspect of integrating AI into clinical workflows is addressing its specific ethical and regulatory challenges. For instance, the use of large datasets, often containing sensitive patient information, raises privacy concerns that require stringent safeguards. Adhering to data protection regulations such as the General Data Protection Regulation (GDPR) in Europe or the Health Insurance Portability and Accountability Act (HIPAA) in the United States is essential.^23^, ^26^ While anonymization and encryption methods can safeguard patient information, achieving true data de‐identification is complex, especially when integrating multi‐modal datasets for AI training and validation.

Regulatory pathways for AI in healthcare further complicate its implementation. AI systems often operate as ‘black boxes,’ providing outputs without transparent insight into their decision‐making processes. This lack of interpretability presents challenges for clinicians who must justify AI‐assisted decisions, particularly in high‐stakes scenarios like CTRCD. Regulatory agencies such as the FDA have begun addressing these issues by developing guidelines for the evaluation of AI‐based medical devices, emphasizing transparency, reliability and explainability.^83^, ^84^ However, the pace of regulatory adaptation often lags behind the rapid evolution of AI technologies, posing challenges in bridging innovation with real‐world clinical implementation. Integrating AI into cardio‐oncology practice also demands careful adaptation of clinical workflows. Many healthcare systems are ill‐equipped to incorporate emerging technologies due to a lack of infrastructure, training and support. Clinicians require robust education on AI tools to interpret outputs effectively and make informed decisions. Ensuring that AI tools are user‐friendly for clinicians is essential to facilitate their adoption in routine practice.^59^

Ultimately, by prioritizing data quality, bias reduction, transparency in algorithm development and ethical considerations, AI can transition from a promising tool to an integral component of cardio‐oncology practice. These efforts have the potential to improve the early detection of cardiotoxicity, optimize therapeutic interventions and support a more personalized approach to managing cardiovascular health in cancer patients.

## CONFLICT OF INTEREST STATEMENT

GZ holds stocks of Immunomica Ltd. and has been consulting for the Menarini Group.

## Data Availability

Data are available upon request and email to GZ at gabriele.zoppoli@unige.it.
